# Retrospective Identification and Characterization of Traumatic Brain Injury—Recommendations from the 2024 National Institute of Neurological Disorders and Stroke Traumatic Brain Injury Classification and Nomenclature Initiative Retrospective Classification Working Group

**DOI:** 10.1089/neu.2024.0590

**Published:** 2025-07-09

**Authors:** John D. Corrigan, Michael L. Alosco, Joukje van der Naalt, Rachel Sayko Adams, Breton M. Asken, Sidney Hinds, Anthony H. Lequerica, Virginia Newcombe, Olli Tenovuo, Eve Valera, Deborah Yurgelun-Todd, Adele Doperalski, Hibah O. Awwad, Kristen Dams-O’Connor, Andrew I.R. Maas, Michael A. McCrea, Nsini Umoh, Geoffrey T. Manley

**Affiliations:** ^1^ Department of Physical Medicine and Rehabilitation, The Ohio State University, Columbus, Ohio, USA.; ^2^ Department of Neurology, Boston University Alzheimer’s Disease Research Center, Boston University CTE Center, Boston University Chobanian & Avedisian School of Medicine, Boston, Massachusetts, USA.; ^3^ University Medical Center Groningen, University of Groningen, Groningen, Netherlands.; ^4^ Department of Health Law, Policy and Management, Boston University School of Public Health, Boston, Massachusetts, USA.; ^5^ Department of Clinical and Health Psychology, Florida Alzheimer’s Disease Research Center, University of Florida, Gainesville, Florida, USA.; ^6^ Department of Radiology/Neurology, Uniformed Services University of the Health Sciences, Bethesda, Maryland, USA.; ^7^ Kessler Foundation, East Hanover, New Jersey, USA.; ^8^ Department of Medicine, University of Cambridge, Cambridge, United Kingdom.; ^9^ University of Turku, Turku, Finland.; ^10^ Harvard Medical School, Massachusetts General Hospital, Boston, Massachusetts, USA.; ^11^ MIRECC George E. Wahlen Department of Veterans Affairs Medical Center, University of Utah, Salt Lake City, Utah, USA.; ^12^ Division of Neuroscience, National Institute of Neurological Disorders and Stroke, Bethesda, Maryland, USA.; ^13^ Department of Rehabilitation and Human Performance and Department of Neurology, Icahn School of Medicine, Mount Sinai, New York, New York, USA.; ^14^ Department of Neurosurgery, Antwerp University Hospital, Edegem, Belgium.; ^15^ Faculty of Medicine and Health Science, Department of Translational Neuroscience, University of Antwerp, Antwerp, Belgium.; ^16^ Department of Neurosurgery, Medical College of Wisconsin, Milwaukee, Wisconsin, USA.; ^17^ Neurological Surgery, University of California San Francisco, San Francisco, California, USA.

**Keywords:** characterization, classification, CBI-M model, framework, medical record extraction, NIH, NINDS, nomenclature, proxy-report, repetitive head impact, self-report, traumatic brain injury

## Abstract

The National Institute of Neurological Disorders and Stroke (NINDS) convened experts in traumatic brain injury (TBI) research, policy, clinical practice and people with lived experience to propose a system of injury classification less susceptible to misinterpretation and misrepresentation inherent in the current use of “mild”, “moderate” and “severe”. One of six working groups addressed Retrospective Classification of TBI. The Working Group consisted of 14 experts in brain injury research representing a breadth of professional disciplines. Initial conclusions based on expert opinion were vetted and revised based on public input at the January 2024 NINDS TBI Classification and Nomenclature Workshop. The Working Group examined five types of methodologies for identifying past TBIs (self/proxy-report, medical record extraction, imaging, fluid-based biomarkers, and performance-based tests). They concluded that self/proxy-report is essential for clinical, research and surveillance applications and that clinicians and researchers should employ elicitation protocols that have been studied and found valid. Medical record extraction was also identified as an invaluable tool for identification of past history of medically attended TBIs; however, there is a need to standardize the case definition employed and procedures used. The use of imaging methods, fluid-based biomarkers, and performance-based assessments in isolation lacked sufficient evidence of both sensitivity and specificity in detecting past histories of TBI to be recommended for this use at this time. The Working Group also evaluated identification of repetitive head impacts (RHI), finding no evidence of a common definition of RHI, a requisite initial step for the development and validation of standardized instruments.

## Introduction

Being able to identify and classify a past history of traumatic brain injury (TBI) is essential for clinical, research, and surveillance purposes. In clinical settings, identification, and classification of prior TBI exposure may inform care management and minimize symptom misattribution. Studies of persons who have a prior history of TBI, including those being served in community-based programs, advance understanding of the late effects of injury. Research using biomarkers or neuropathology to understand the biological correlates of TBI also requires accurate classification of TBI history. In surveillance, having a method for capturing TBI history is critical for accurately measuring the public health burden of these injuries.

**FIG. 1.**
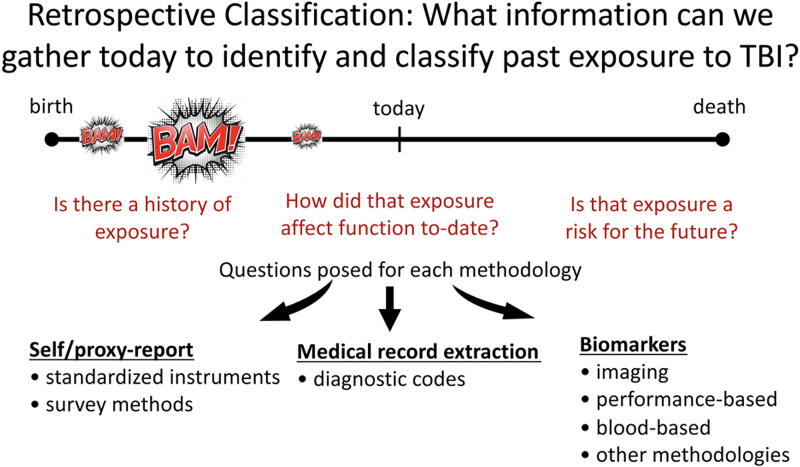
Retrospective classification: What information can we gather today to identify and classify past exposure to TBI? This figure depicts the three questions (in red font) that were posed for each methodology examined. The timing in a person’s life course that is pertinent to each question is shown above the questions and the methodologies examined are shown below. The arrows depict that the three questions were asked for each methodology. TBI, traumatic brain injury.

The National Institute of Neurological Disorders and Stroke (NINDS) convened experts in TBI research, policy, and clinical practice as well as people with lived experience to propose a system of injury classification that is not susceptible to misinterpretation inherent in the current use of “mild”, “moderate” and “severe”. Six working groups met during 2023, culminating in recommendations vetted at the NINDS TBI Classification and Nomenclature Workshop in January 2024. One of the working groups addressed Retrospective Classification, evaluating the current state of the science and practice when using a broad array of methodologies (self/proxy report, medical record extraction, imaging, fluid-based biomarkers, and performance-based biomarkers) to detect and characterize prior exposure to TBI. For each methodology, the Working Group addressed the extent to which it can validly (1) detect a history of prior exposure to TBI, (2) characterize whether/how that exposure affected past and current functioning, and (3) predict risk for future consequences of past exposure (see Fig. 1).

For each methodology evaluated, the Working Group also sought to address exposure to repetitive head impacts (RHI). Our working definition of RHI was an environmental exposure to repeated hits, impacts, blows, or forces to the head regardless of whether a diagnosed or known TBI occurred. Exposure to RHI is often assessed by querying an individual’s history of participation in specific activities such as contact and collision sports or military duties, regardless of the effect on brain function. Sub-concussive injuries are often discussed in the context of RHI; however, consensus definitions of these injuries, as well as methods for their accurate estimation, are nascent.

The scope of the work assigned to the Retrospective Classification Working Group was initially proposed by the Workshop Steering Committee as part of the wider goals of the NINDS TBI Classification and Nomenclature Workshop. Conclusions were reached based on expert opinion. The Working Group consisted of 14 experts in brain injury research representing a breadth of professional disciplines. The Working Group is divided into subgroups composed of Working Group members with specific expertise on the methodology addressed. Subgroups met from July 2023 through September 2023 and brought back conclusions and recommendations to the full group. A summary of the Working Group’s conclusions was circulated among all six of the Classification and Nomenclature Working Groups and revised in response. The resulting recommendations were subsequently shared with attendees at the January 2024 Workshop held in Bethesda, Maryland. The final recommendations, described here, resulted from discussion at the Workshop.

## Self/Proxy-Report via Standardized Instruments

There are several standardized instruments that have demonstrated criterion-related and construct validity,^1–9^ though only some have been tested for reliability.^1,2,4,5,8,10–12^ Capturing the lifetime history of TBI exposure employing self-report instruments should use these standardized and validated instruments for both clinical and research purposes. Most instruments use a two-tiered approach of first identifying events that may have resulted in an external force applied to the brain, followed by elicitation of the nature of altered brain function (e.g., being dazed, having a gap in memory, loss of consciousness) arising from that event. It is strongly recommended that respondents should not be asked to self-diagnose by using terms like “concussion” or “traumatic brain injury” in elicitation, as this approach is more prone to bias based on respondents’ differing knowledge and understanding of these terms.^4,13^

All standardized instruments were developed for use as a contemporaneous interview, though the Ohio State University TBI Identification Method^2,14,15^ and the Brain Injury Screening Questionnaire^3,7^ also have self-administered versions. It is presumed that self-report can be used with adults and adolescents who are capable of understanding the questions though there has been no reported testing of the youngest age at the time of interview for which use is valid. For children and younger adolescents, a proxy report by a parent or other adult who has a thorough knowledge of their exposure history should be employed. While most instruments address lifetime exposure to TBI, some have a narrower scope that focuses on a particular population (e.g., military service members^5,6^ or victims of domestic violence^16^), type of exposure (e.g., blast-related^12^), or intended use (e.g., symptomology that may become the target of treatment^13,15^). Instruments should be used for the purpose for which they were developed and validated.

Many studies using standardized instruments indicate that injury severity as manifested by the extent of altered brain function at the time of injury has the greatest predictive and prognostic value.^7,17–21^ Self-reported loss of consciousness, especially with longer duration, is associated with greater deficits in neurobehavioral function.^22–25^ There is also support for a history of multiple TBIs, regardless of the severity, to confer greater neurobehavioral consequences.^16,26–28^ There is some indication that age at injury and injury characteristics may interact, specifically less severe childhood injuries manifesting in adult neurobehavioral consequences.^29,30^

Finally, exposure to RHI from contact and collision sports and other sources captured via self-report instruments has been associated with long-term neurological and neuropsychiatric outcomes.^31^ However, most of the literature is based on male former elite contact sports participants. More research is needed for all causes of RHI on the ability of standardized instruments to characterize past effects, current functioning, and/or future prognosis.

## Self/Proxy-Report via Survey Methods

Self/proxy reports incorporated into survey methodologies provide a less time-consuming alternative to using standardized instruments and are particularly useful for estimating TBI incidence and prevalence. These methodologies also provide an alternative to health care administrative datasets for population estimates by capturing those who sustained a TBI but did not seek care^32^ or sought care but did not receive a diagnosis.^33–36^ In recent years, researchers have used a wide variety of approaches to add questions to national surveys to better quantify the burden of TBI. Each approach has benefits and drawbacks.

Self-reporting is preferred over proxy-reporting when there is a choice.^37,38^ Generally, individuals are able to report more accurately on their own experiences. However, in the case of children or severely impaired adults, it is necessary to rely on proxy reporting. Due to issues with recall bias and telescoping, eliciting reports of more recent events will likely be more accurate than reporting on lifetime experiences. However, surveys may need to ascertain lifetime prevalence in order to capture respondents’ full exposure to TBI or attain sufficient numbers for analysis. Utilizing a series of questions, versus a single question, allows for a more inclusive elicitation of TBI and for the possibility of categorizing injuries based on the certainty that they constituted a TBI (e.g., probable vs possible, or diagnosed vs suspected TBI).^39–41^ Using multiple questions also allows survey administrators to better frame the injury, either by defining TBI or spurring recall by providing examples of how the respondent might have been injured. Requiring a specific diagnosis to be reported (e.g., “has a doctor ever told you that you had a TBI?”) will result in under-identification and is not recommended on self-report surveys. Researchers need to be thoughtful about the terminology used in self-report surveys; asking about a “head injury” will likely elicit a different response than a “traumatic brain injury.” Using the term “concussion” or “TBI” will be limited by the accuracy of respondents’ knowledge of these terms as well as the actual terminology used by medical professionals,^13,42^ which could vary by region or country.

Future research is needed to determine the accuracy of incidence and prevalence estimates derived from self-reports in survey methodologies. There is currently no gold standard method to use as a comparator.^43^ With few exceptions, most of the research on self-reporting has been limited to English-speaking respondents—international and cross-cultural studies should be undertaken to assure generalizability of these methods. Efforts should be made to establish best practices for eliciting RHI via self-report and survey methodologies.

## Medical Record Extraction

Medical record extraction is an invaluable tool in population research for the identification of a past history of *medically attended* TBI. Methods for abstracting a documented occurrence of TBI in an electronic medical record typically rely on International Classification of Diseases, Tenth Revision, Clinical Modification (ICD-10-CM) coding but may include documented clinical signs, acute symptoms of TBI, or, when available, abnormal findings from contemporaneously captured biomarkers.^44,45^ In addition to knowing that many do not seek medical attention for TBI, potential weaknesses of medical record extraction include absent or inaccurate documentation, lack of specificity of symptoms, loss of information due to change of residence or health insurance, and limitations to proper coding of the injury.^34,46–49^

Several issues affect the validity of TBI diagnoses captured from medical records. The certainty of diagnosis from medical record extraction of more severe TBI is less problematic than for less severe TBI as symptoms for the latter may be unnoticed or nonspecific. A limitation of medical record diagnosis coding is the differentiation of new injuries from previous ones and the inability to capture RHI. The ability to identify the consequences of TBI is difficult as functioning in a variety of domains (e.g., resumption of social roles) is often not documented.^50^ Long-term effects, including those based on assessments like neuropsychological testing, may not be routinely captured in the medical record.^45,50,51^ Without documentation of later effects, it is unlikely information can be extracted from medical records about how prior exposure to TBI affects current functioning.^52^

There are several gaps in knowledge related to the use of medical records to retrospectively identify TBI. First, multiple diagnostic codes are used to denote TBI and there is no consensus on which algorithm(s) constitute best practice. Uncertainty regarding the use of S09.90, as well as unspecified injury of the head, is an example.^46,49^ Furthermore, the use of multiple algorithms limits comparisons across studies. Second, there is no research showing whether care providers or coders in different medical settings use appropriate or consistent codes for TBI. Third, current coding practices do not enable the determination of multiple TBIs (e.g., new injury vs treatment for prior injury), or the presence of RHI. Finally, current practices do not provide information on the long-term effects of TBI. Recommendations for future directions include the establishment of expert consensus on the use of diagnostic coding for TBI. Research is warranted on artificial intelligence and machine learning techniques that could retrospectively identify TBI cases in medical records. Additional training of medical care providers and coders is needed. Finally, to limit discrepancies between actual versus diagnosed cases, additional investigation is needed on the documentation of clinical variables used to ascertain TBI.

## Imaging

As there was another working group focusing on imaging including the acute phase, the Retrospective Working Group examined imaging in relation to chronic changes. Several neuroimaging modalities are used in clinical care of TBI as well as in ongoing TBI research including but not exclusive to computed tomography (CT), magnetic resonance imaging (MRI), transcranial Doppler, positron emission tomography (PET), single photon emission computed tomography (SPECT), and functional near-infrared spectroscopy.^53^ Cranial CT scanning is primarily used in the acute phase of head injury to diagnose focal injuries and guide neurosurgical interventions. However, CT imaging has less utility in detecting the often subtle changes in chronic stages of TBI and is rarely included in research studies outside of the acute injury.^54^ MRI techniques do not have ionizing radiation and are more readily available for implementation than PET or SPECT that require radiotracers.^55^

MRI is widely used in clinical and research applications and is more sensitive to structural injuries in the brain, particularly damage to the white matter.^56,57^ While quantitative MRI findings are not typically used to guide clinical care, various types have been used to examine brain changes related to both acute and chronic TBI with quantitative analysis of sequences including T1-weighted, diffusion-weighted, and resting-state functional imaging being particularly useful to understand the longer-term effects over the more clinical sequences.^58–62^ MRI methods may effectively detect past TBI-related changes in brain structure, function, and neurochemistry that may predict functional outcomes.^63,64^

MRI findings indicating post-injury neurodegenerative changes that occur because of TBI include progressive atrophy of the brain and reduced white matter integrity.^59,61,62,65^ Interestingly, in the chronic stages of TBI, dynamic quantitative structural changes have been shown to happen concurrently with behavioral recovery,^62,66,67^ and this may be an important window for interventions.^68–70^ However, little work has been done to understand the recovery processes occurring during this time period. MRI imaging approaches may be useful to parse out beneficial and deleterious brain changes during the chronic stages of TBI and may uncover potential avenues of therapy that minimize deleterious changes while maximizing beneficial ones.^71^ Interpretation of findings to date should be considered in light of imaging methods and analyses, selection of populations studied (e.g., civilians, military, or athletes), potential comorbidities including substance use and psychiatric disorders, and other potentially important modifiers.^72^

A wide array of other neuroimaging and neurophysiological approaches, including PET, SPECT, EEG, qEEG, and MEG, have also been used to examine chronic post-TBI effects in the brain; however, a review of these methods was beyond the scope of this project but should be addressed in future refinements of classification approaches.^53^

## Performance-Based Measures

Performance-based evaluations like neuropsychological assessments currently lack specificity to the effects of a past TBI or RHI particularly when interpreted in isolation.^73–77^ Self-report or medical record extraction will be necessary for the identification of the occurrence of a past TBI or RHI. Performance-based evaluations alone are insufficient for the determination that a past TBI or RHI occurred but may help inform whether the identified history of TBI or RHI is contributing to current symptoms.^78^ To attribute symptoms to a past TBI, it is necessary to rule out other sources of central nervous system compromise that may be affecting objective findings or subjective reports. Determining the extent to which the results of a performance-based evaluation can be attributed to past TBI or RHI depends in part on the temporal relationship between the event that could cause symptoms, the types of symptoms, and the frequency, severity and/or developmental stage at the time of the TBI exposure, as well as lack/presence of other potential explanations. Confidence in concluding that current symptoms are due to past TBI increases with injury severity and shorter intervals between TBI and the performance-based evaluation.

It is possible that a *later life* TBI can be superimposed on an underlying neurodegenerative disease process, worsening or unmasking the disease process even if the individual was asymptomatic prior to the injury (i.e., rather than *causing* the underlying disease *per se*), or could contribute to variability in age of symptom onset,^79–81^ types of symptoms experienced,^82–84^ or rate of progression.^85^ The presence of chronic traumatic encephalopathy (CTE) at autopsy strongly implicates exposure to RHI. However, the specific clinical correlates and profiles of CTE, as well as non-CTE neuropathologies that might arise from RHI remain an area of active investigation. Future research should seek a better understanding of the relative contributions of TBI and RHI to the biological and clinical features of neurodegenerative disease processes. Integrating comprehensive neuropsychological and neurological evaluations with increasingly available multimodal biomarker measurement(s) is expected to rapidly accelerate our understanding of the spectrum of contributions of lifetime TBI and RHI to current and future brain health.

## Blood-Based Biomarkers

At the current nascent stage of investigation, there is insufficient evidence for the use of blood-based biomarkers to detect past histories of TBI. However, there are now examples where blood-based biomarkers are rapidly moving toward clinical applications in the diagnostic workup of older adults with cognitive and behavioral concerns. Proteins measurable in blood range from nonspecific indicators of neurodegeneration (e.g., neurofilament light chain, NfL) to disease-specific markers of Alzheimer’s disease (AD) like plasma p-tau217.^86,87^ There are currently no blood-based biomarkers that signal neurodegenerative effects specific to prior TBI or RHI, such as CTE or TBI-related forms of neurodegeneration. Nonspecific neurodegenerative proteins like plasma NfL and total tau have been reported in a few studies as modestly elevated in older adults with a history of TBI,^88,89^ and NfL levels in the early chronic phases have been found to correlate with the annual atrophy rate years after injury.^90–92^ Current possible contexts of use for blood-based markers in clinical research of adults with and without TBI include characterizing nonspecific neurodegenerative and potential neuroinflammatory processes (e.g., NfL, glial fibrillary acidic protein) or establishing the presence of AD pathology (p-tau217) to contextualize possible contributors to cognitive or behavioral changes irrespective of the specific role of prior head trauma. Using available validated biomarkers to rule out AD pathology may also facilitate differential diagnosis from competing neurodegenerative diseases.^87,93,94^

## Discussion

The Retrospective Working Group identified priorities for immediate translation into research and practice (see Table 1) as well as issues requiring additional research (see Table 2). Given the impetus to re-examine nomenclature and characterization of TBI, the Working Group concluded that in the absence of sufficient evidence for classifying the effects of retrospectively identified TBIs, the terms “mild,” “moderate,” and “severe” should not be used. Instead, past TBI characterization should be based on the method used to identify a past injury (e.g., “contemporaneous medical record documentation,” “self-reported loss of consciousness”) until such time as there is sufficient evidence to classify past TBIs by their prognostic implications.

**Table 1.** Retrospective Working Group Translational Priorities

1. In the absence of sufficient evidence for classifying the effects of retrospectively identified TBIs, the terms “mild”, “moderate” and “severe” should not be used in research or clinical applications. In lieu of these terms, characterization of past TBIs should refer to the method used to identify injury (e.g., “as indicated by contemporaneous medical record documentation”, “self-reported loss of consciousness”).
2. Self/proxy-report methodologies a. There has been sufficient study of both standardized instruments and survey methodologies for self/proxy-report that whenever possible clinicians and researchers should employ elicitation protocols that have been studied and found valid, whether those existing or newly developed. b. Two-tiered self/proxy-report elicitation approaches that separately identify events involving external forces and the effects of the events on brain function are superior to approaches that use diagnostic terms like “traumatic brain injury” or “concussion” to elicit recall.
3. Medical record documentation a. Clinical, biomarker, and imaging information, as well as relevant modifiers, needed for characterization of acute TBI should be available in the medical record for extraction. b. Best practices for design of medical record extraction studies should be developed. c. Coding schemes for medical record extraction should be elaborated to better detect follow-up care, late effects of TBI, and multiple TBIs. d. New developments in clinical, biomarker, imaging, and modifier characterization should be integrated into future ICD revisions. e. International agreement on coding is needed. f. Training and guidance for providers and coders should be expanded to improve the quality of medical record documentation.
4. Methods of characterizing acute TBI are not sufficiently sensitive and specific to be used to identify past TBI. a. Imaging methods, particularly MRI, are specific but not sensitive to detection of prior TBIs. b. Blood-biomarkers of past TBI are in a nascent stage of investigation and cannot yet be used for detection and characterization of past TBI. c. Performance-based methods in isolation do not have sensitivity and specificity to detect *prior history* of TBI but are useful for characterizing the effects of prior injuries.
5. Guidance for characterizing a past history of TBI, and its effects, should be developed for clinical use.

TBI, traumatic brain injury; ICD, international classification of diseases; MRI, magnetic resonance imaging.

**Table 2.** Retrospective Working Group Research Priorities

1. Convene a consensus conference to establish a precise case definition of ICD-10 codes to be used in medical record extraction. New concepts for characterization of TBI should be integrated into these deliberations, as possible.
2. Convene a consensus conference to establish a case definition of RHI. A consensus definition is required as a precursor to development or refinement of valid and standardized measures.
3. Additional research is needed on the following issues: a. The use of imaging in the chronic phase post-injury to characterize and classify past TBI. b. The use of blood-based or other fluid biomarkers in the chronic phase post-injury to characterize and classify past TBI. c. Development and validation of self/proxy-report in unique populations, elicitation settings, non-English speaking respondents, and diverse cultural contexts. d. The limits on accuracy of self/proxy-report due to factors such as age at injury, remoteness of the event, TBI severity, and characteristics of the reporter (e.g., proxy or self-report, current cognitive or emotional status, or other sources of reporting bias). e. The prognostic value of self/proxy-reported history of TBI. f. Instrument development and validation for the retrospective identification of RHI in multiple settings and uses.

TBI, traumatic brain injury; RHI, repeated head impacts.

Advances in the classification of TBI by acute clinical, biomarker, and imaging characteristics, with salient modifiers, should eventually allow greater accuracy in characterizing past TBIs. However, there are uses for eliciting a lifetime history of TBI that may not fully align with models developed for acute injury characterization which focuses on an index injury, in most cases, recently incurred. Past TBI identification is also needed for estimating population prevalence, understanding the public health burden, or predicting vulnerability to an additional TBI or other adverse health outcomes. These uses require the characterization of lifetime exposure and not just an index injury. For instance, to fully characterize toxic exposures requires knowledge of the number, duration, and developmental stage that a person was exposed to a toxin.^95^ When a full picture of TBI exposure is needed, the salient parameters would differ from those used to characterize an index injury.

Self/proxy reports, whether via standardized instruments or survey methodologies, are essential for clinical, research, and surveillance applications, providing information that cannot be obtained via other methods. There has been sufficient study of both standardized instruments and survey methodologies that whenever possible clinicians and researchers should employ elicitation protocols that have been studied and found valid. Continued development and validation should be directed to unique populations, elicitation settings, non-English-speaking respondents, and diverse cultural contexts. Two-tiered elicitation approaches that separately identify events involving external forces and the effects of the events on brain function are superior to approaches that use the terms “traumatic brain injury” or “concussion” to elicit recall. More research is needed to (1) understand the limits on accuracy of self-report due to age at injury, remoteness of the event, TBI severity, and characteristics of the reporter (e.g., proxy or self-report, current cognitive or emotional status) and (2) better characterize the prognostic value of exposure history when elicited via self/proxy-report.

Medical record extraction is an invaluable tool in population research for identification of past history of *medically attended* TBI. However, there is an immediate need for consensus on a case definition. Coding schemes should be elaborated to better detect follow-up care, late effects of TBI, and multiple TBIs. Training for providers and coders should be expanded to improve the quality of documentation.

Imaging or fluid-based biomarkers as well as performance-based assessments lack sufficient evidence of sensitivity and specificity in detecting past histories of TBI to be recommended for this use at this time. Imaging methods, particularly MRI, are specific but not sensitive to the detection of prior TBIs. Features described include (but are not exclusive to) gliosis, atrophy (which may be generalized and or in specific areas e.g., hippocampus), and a cavum septum pellucidum. More advanced quantitative methods provide some insight into residual effects, but more research is needed to fully investigate the utility of these techniques when identifying remote injuries. The prognostic value of imaging of past TBIs has not been established. Blood-based biomarkers of past TBI are in a nascent stage of investigation. The Working Group did not attempt to address other methodologies including electrophysiologic-based biomarkers, which should be undertaken in future iterations of this work. The accuracy of performance-based methods is limited by both sensitivity and specificity. There is not a performance-based assessment that is specific to the *prior history* of TBI, but they are still important tools for an individual clinical assessment.

Research and development will be needed to inform best practices for the retrospective identification of RHI. The establishment of a consensus case definition should be pursued as a precursor to the development or refinement of valid and standardized measures. Currently, the concepts of sub-concussive blows to the head and multiple mild TBIs (which by definition do not overlap) are not consistently included or excluded when operationalizing RHI.

## Conclusion

The Retrospective Classification of TBI Working Group found that only two (self/proxy-report and medical record abstraction) of the five methodologies examined had sufficient research to recommend use for characterizing the prior history of TBI. Both of those methodologies will benefit from additional research and, in the case of medical record abstraction, consensus regarding a case definition and standardization of recording. There was sufficient evidence from studies of self/proxy reports elicited via both standardized instruments and survey methodologies to recommend that clinicians and researchers employ protocols that have been found valid. The use of imaging methods, fluid-based biomarkers, and performance-based assessments in isolation lacked sufficient evidence of *both* sensitivity and specificity in detecting past histories of TBI to be recommended for this use at this time. There was no evidence of a common definition of RHI, which must be addressed to allow the development and validation of standardized instruments. Despite the shortcomings identified by the Working Group, there was strong support for continuing to study and refine methods of characterizing past TBI and RHI as this capability is critical for clinical, epidemiological, and research applications.

## Transparency, Rigor, and Reproducibility

This article did not involve the analysis of original data.

## Disclaimer

The views expressed in this article are those of its authors. S.H.’s views should not be viewed as the opinion or official policy of the Uniformed Services University, Defense Health Agency, Department of Defense, or the Federal government. The content is solely the responsibility of the authors and does not necessarily represent the official views of the National Institute of Neurological Disorders and Stroke, the National Institutes of Health, and the U.S. Department of Health and Human Services.
